# Advances in Modern Structural Engineering: From Materials to Building Structures

**DOI:** 10.3390/ma19051037

**Published:** 2026-03-09

**Authors:** Zhihua Chen, Yiyi Zhou, Hongbo Liu, Hai Zhang

**Affiliations:** 1 Department of Civil Engineering, Tianjin University, Tianjin 300072, China; 2 College of Future Technologies, Hohai University, Changzhou 213251, China; 3 School of Civil Engineering, Hebei University of Engineering, Handan 056038, China; 4 Department of Civil Engineering, Tianjin Chengjian University, Tianjin 300384, China

## 1. Introduction

Structural engineering is currently at a critical stage of development, driven by growing global demands for sustainability, resilience, and construction efficiency. Traditional structural paradigms, which have long been constrained by conventional materials and labor-intensive construction methods, are undergoing a profound transformation. This shift is being driven by both the rapid development of advanced materials and the reimagining of structural systems themselves. This Special issue outlines the major trends in this field and discusses progress in the future built environment on the basis of the deep integration of novel materials, including ultra-high-performance composites, smart responsive materials, and bio-based materials, as well as innovative structural forms such as modular assemblies, advanced steel systems, and composite construction. Such integration not only enhances structural performance but also represents a key step toward achieving sustainability under the pressures of climate change, dynamic loading, and rapid urbanization.

Advancements in materials science have created unprecedented opportunities for structural engineering [1,2,3,4]. The emergence of new materials has removed the performance limitations of traditional options, providing engineers with a richer set of design possibilities. Ultra-high-performance concrete (UHPC) and high-strength low-alloy steels, for instance, have significantly improved structural strength, durability, and span capacity, enabling more lightweight components with enhanced life-cycle performance [5,6]. At the same time, the development of smart materials—such as shape memory alloys, piezoelectric materials, and phase-change materials—has endowed structures with sensing, response, and self-monitoring capabilities, laying the foundation for adaptive and self-regulating structural systems [7]. Driven by the need for carbon reduction, bio-based materials such as cross-laminated timber (CLT) and bio-composites have gradually become important alternatives to high-carbon materials, promoting the integration of structural engineering with ecological and circular economy principles.

Under the impetus of material innovation, the design philosophy of structural systems is also evolving. Prefabrication and modular construction have significantly improved construction efficiency, quality control, and resource utilization through factory-based production and rapid on-site assembly [8,9]. Advanced steel structures and composite systems—including steel–concrete composite (SCC), timber–concrete composite (TCC), and textile-reinforced concrete (TRC)—enable performance optimization through the synergistic interaction of multiple materials, providing more efficient solutions for super-tall buildings and long-span structures [10,11,12]. The development of bio-based materials has further encouraged the exploration of new low-carbon structural systems, such as tall timber buildings and bio-composite components, offering new pathways for the sustainable transformation of the construction industry [13].

The deep integration of material innovation and structural system reform is the core driving force behind the development of modern structural engineering. Smart materials provide the functional basis for new structural systems, while innovative structural forms promote further improvements in material performance. For example, modular bio-composite units integrated with sensors can be used to construct highly efficient adaptive structures, and UHPC precast bridge decks prestressed with carbon fiber tendons exemplify the synergistic benefits of high-performance materials and advanced construction technologies [14,15,16]. This interdisciplinary integration is pushing structural engineering beyond traditional mechanics-based analysis toward a more holistic approach that combines materials science, digital fabrication, intelligent systems, and ecological design.

Despite the promising prospects of new materials and structural systems, research in this field still faces challenges such as incomplete regulatory frameworks, difficulties in large-scale material production, and the lack of comprehensive life-cycle assessment methods. Nevertheless, the overall trend indicates that future building structures will be more resource-efficient, intelligent, resilient, and environmentally friendly.

This Special Issue aims to collect and disseminate the latest research progress in structural engineering, promote the innovative development of new materials and structural systems, and provide academic support for achieving safer, more efficient, and more sustainable built environments.

## 2. Overview of the Published Articles

A total of five articles have been published, which cover the following different aspects of novel materials and innovative structural forms used for buildings: the durability of graphene oxide concrete composite, the properties of basalt fiber-modified phosphogypsum planting concrete, a review of the mechanical behavior and performance degradation of structural cables, the load-bearing capacity of a thin-walled perforated beam accompanied with chipboard panels, and an enhanced joint integrity assessment in steel structures. All of these aspects provide advanced insights in this field.

The durability of graphene oxide (GO) concrete composite under combined chloride and sulfate environments was studied by Gao et al. (Contribution 1). Specimens containing 0.07% GO by weight were subjected to both dry–wet cycling and long-term immersion in a mixed solution. The analysis focused on evaluating performance degradation through compressive strength tests, mass loss measurements, stress–strain relationship analysis, and scanning electron microscopy (SEM) for microstructural examination. The results show that the 0.07% GO dosage optimally enhanced durability, demonstrate by the minimal strength reduction and mass loss, improved ductility post-corrosion, and a denser, more regulated microstructure that effectively delayed degradation mechanisms.

The mechanical- and permeability-related properties of phosphogypsum-based planting concrete following modification with basalt fibers were investigated by Zhang et al. (Contribution 2). Compressive strength, porosity, and sand permeability were evaluated through a series of laboratory tests. The results indicate that the incorporation of basalt fibers effectively improved compressive strength, with longer fibers (18 mm) contributing to a more pronounced enhancement than shorter fibers (6 mm). Meanwhile, an increase in fiber content led to a gradual decrease in porosity. The addition of basalt fibers also reduced both the sand permeability and water permeability coefficient. Specimens containing 6 mm fibers exhibited a greater reduction in permeability than those with 18 mm fibers. Furthermore, higher fiber content significantly enhanced the water retention capacity. These findings provide a theoretical basis for the design and optimization of fiber-reinforced planting concrete for ecological engineering applications.

The mechanical properties, degradation mechanisms, and post-fracture behavior of major cable types used in building structures, such as semi-parallel wire strand (SPWS), Galfan-coated steel strand (GSS), and full-locked coil wire rope (LCR), were reviewed by Chen et al. (Contribution 3). The discussion focuses on five critical aspects: fundamental cable characteristics, stress relaxation and creep, mechanical performance under high temperatures, corrosion-induced degradation, and behavior after fatigue-induced wire breaks. Key mechanical parameters including elastic modulus, axial stiffness, bending stiffness, and the coefficient of thermal expansion were identified. The results highlighted the superior corrosion resistance of LCR and GSS and elucidated the redistribution of stress and residual capacity following the rupture of steel wires. Based on recent studies, the authors suggested prospective research directions to address current knowledge gaps and advance durability-focused design strategies for future cable-supported structures.

Based on finite element (FEM) simulations, the load-capacity curves and failure modes of the central beam within a structural assembly comprising thin-walled perforated steel beams and a particleboard panel were investigated by Denisiewicz et al. (Contribution 4). Both physical and geometric nonlinearities, as well as detailed contact interactions, were incorporated into the numerical model, and the simulations spanned systems with beam lengths ranging from 3 to 6 m. To verify the model’s reliability, laboratory tests were conducted on two representative configurations with spans of 3 m and 6 m, and the mechanical properties of the beam materials were evaluated using samples extracted from the tested elements. The experimental results confirmed the accuracy of the numerical model, validating its suitability for analyzing the structural response across the considered span range.

The efficacy of a novel pulsed electromagnetic field (PEMF) device for dynamic testing and structural health monitoring was investigated by Mironovs et al. (Contribution 5). The device, comprising a PEMF generator and a flat multifilament coil, was evaluated on a model steel stand with two joint configurations. Analyses of the oscillation pattern and spectral characteristics demonstrated the device’s ability to differentiate between joint states. A 15% reduction in high-frequency components was observed in the 4 mm plate configuration compared to the 8 mm plate. For 3D-printed specimens, fundamental resonant frequencies near 5100 Hz with Q-factors between 200 and 300 were identified, and a 10% increase in volumetric porosity was found to cause a 7% downward shift in resonant frequencies. When integrated with the coaxial correlation method, the system exhibited enhanced sensitivity for detecting structural changes. This integrated approach offers a 30% improvement in early-stage degradation detection compared to traditional methods.

## 3. Conclusions and Outlook

Driven by the need to meet escalating global demands for enhanced sustainability, operational resilience, and construction efficiency, the field of structural engineering is undergoing a profound paradigm shift. This transformation is fundamentally anchored in the synergistic integration of next-generation materials with radically innovative structural systems and construction methodologies. The selected studies presented in this Special Issue encapsulate the research at the forefront of these developments. This collection offers a critical summary of the latest theoretical, experimental, and applied advances, providing researchers, designers, and practicing engineers with indispensable insights for pioneering the innovation, optimization, and practical implementation of advanced materials and structural forms.
